# Replantation of Teeth

**Published:** 1874-05

**Authors:** 


*Replantation of Teeth.*—From the *London Lancet* we take the following:

"Replantation of teeth for acute and chronic peridontitis was suggested by Mr. Coleman after seeing the same remedy succeed for acute inflammation of the pulp of a lower molar tooth, which had resisted every known kind of treatment. The principal objection urged against replantation of teeth is that if a tooth is extracted it must necessarily loose its vitality, and therefore the fangs undergo absorption, so that after a time it becomes useless and must be extracted. Supposing the objection to be valid, as absorption is a long process, sometimes extending over years, it will have been a greater gain for a patient to retain his tooth for an indefinite period than to lose it entirely and at once; but it is no more necessary that a tooth, after undergoing extraction and replantation, should lose its vitality than for a long bone to do so after fracture, with stripping back of the periosteum.

The manner of performing the operation is as follows: A tooth which is to be replanted should be carefully extracted, and as little as possible of the surrounding tissues lacerated. It should then, unless the operation be simply for the destruction of the dental pulp, and where the periosteum is healthy, be immersed in some antiseptic fluid, such as diluted carbolic acid or chloride of zinc (the latter from experience being preferred.) The socket should then be swabbed out some half dozen times with a strong solution of the same antiseptic employed. The tooth, if carious, should be plugged and returned to its place. If there is any thickening of periosteum, fibrous growth, sac of abscess, or absorption at extremity of fang, it should be excised before replantation. Should the patient complain of pain arising from the operation, prescribe poppy fomentations, although the pain is rarely more than what is due to the tenderness of parts from laceration of soft tissues after the extraction of the tooth.

Out of twelve cases that Mr. Coleman has operated on within the last four years, nine are successful and three have failed. The failures have but one significance, and that is, teeth to undergo replantation must be selected. In a cachectic patient the chances are against success; when a tooth has lost the support of its fellows on both sides it cannot become firm. Nevertheless the successful cases warrant a further trial of replantation, which would preserve many teeth otherwise sacrificed."

